# Bacterial Immune Evasion through Manipulation of Host Inhibitory Immune Signaling

**DOI:** 10.1371/journal.ppat.1004644

**Published:** 2015-03-05

**Authors:** Kristof Van Avondt, Nina M. van Sorge, Linde Meyaard

**Affiliations:** 1 Laboratory of Translational Immunology, Department of Immunology, University Medical Center Utrecht, Utrecht, The Netherlands; 2 Department of Medical Microbiology, University Medical Center Utrecht, Utrecht, The Netherlands; University of North Carolina at Chapel Hill School of Medicine, UNITED STATES

An innate immune response is essential for survival of the host upon infection, yet excessive inflammation can result in harmful complications [1]. Inhibitory signaling evolved to limit host responses and prevent inflammatory pathology [2,3]. Given the significance of inhibitory pathways for immunity and homeostasis, they provide ideal targets for manipulation by bacterial pathogens. Recent evidence highlights that bacteria have developed diverse strategies to exploit these inhibitory pathways to avoid host defense for their own benefit. In this review, we cover these different immune evasion strategies for the first time. The recent literature discussed emphasizes that bacteria subvert host immune responses not only by direct engagement of inhibitory receptors (i.e., often through “molecular mimicry” of host ligands [4,5]) but also through virulence factors that resemble intermediates of host inhibitory signaling and interfere with defense functions [6–8]. Understanding how bacteria manipulate inhibitory signaling affords promising opportunities to counteract these escape strategies and tip the balance in favor of the host. In addition, these understandings may provide useful insights on the functional roles of inhibitory pathways in limiting host responses and preventing pathology.

## Inhibitory Signaling Controls Inflammatory Responses in Host Immune Cells

**Fig 1:**
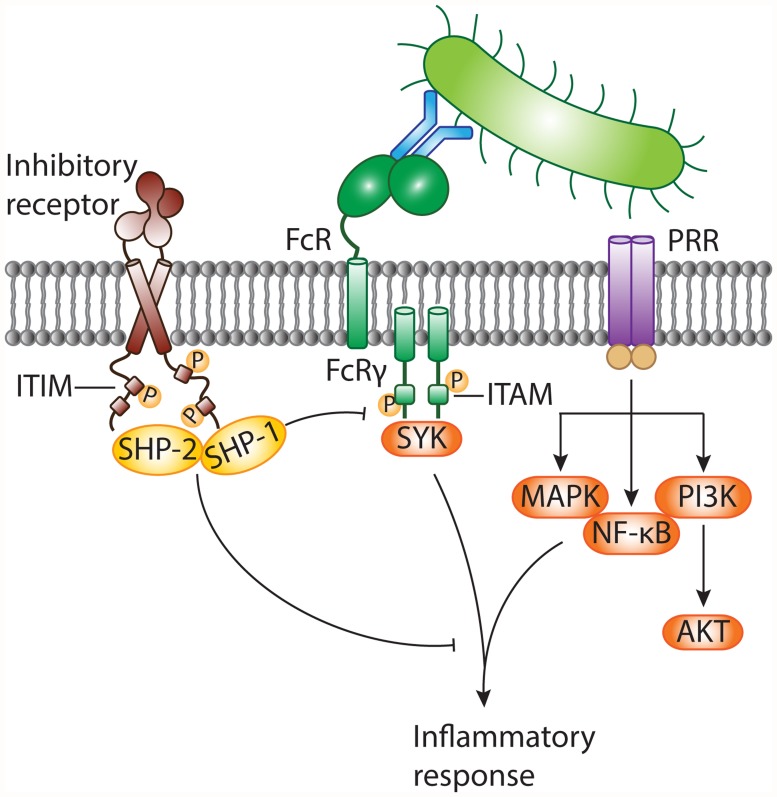
Negative modulation of inflammatory responses against pathogens by ITIM-bearing inhibitory receptors. Invasion of the host by bacteria results in the appearance of pathogen-associated molecular patterns (PAMPs). These danger signals are sensed by pattern recognition receptors (PRRs), including TLRs, on the surface of sentinel cells. Bacteria can be opsonized with antibodies and are recognized by cell surface Fc receptors (FcRs) associated with the immunoreceptor tyrosine-based activation motif (ITAM)-containing FcR common γ chain. FcRs generally transmit activating signals through activation of the protein tyrosine kinase SYK, while diverse signaling cascades (such as activation of MAPK, NF-κB, and PI3K) are relayed by PRRs. The inflammatory response against non-self is essential to combat invading bacteria. On the other hand, the antibacterial response needs to be controlled to prevent collateral tissue damage. Inhibitory receptors often possess immunoreceptor tyrosine-based inhibitory motifs (ITIMs) within their intracellular tails. Following receptor engagement, tyrosine residues within the ITIMs are phosphorylated and become docking sites for cytosolic protein tyrosine phosphatases, such as SHP-1 and SHP-2. These negative regulatory proteins terminate activating signals delivered by PRRs and/or ITAM-coupled FcRs and contribute to dampening of the inflammatory response. MAPK, mitogen-activated protein kinase; NF-κB, nuclear factor κB; PI3K, phosphoinositide 3-kinase.

In response to infection, the host immune system initiates swift and robust inflammatory responses to protect the host from the spread of invading microbes. Inflammation is launched when front-line defense cells, such as epithelial cells, macrophages, and neutrophils, detect alarm signals. The sensing of microbes through pattern recognition receptors (PRRs) activates the inflammatory functions of sentinel cells. However, if the initial host response is overamplified, inflammation results in host tissue damage and can lead to severe complications. Inhibitory pathways control host immune responses upon infection and prevent collateral tissue damage. Inhibitory immune receptors attenuate cellular signaling delivered by activating receptors, including Toll-like receptors (TLRs) [3] and Fc receptors (FcRs), directly or through their downstream signaling intermediates (see review [9] and Fig. 1). Inhibitory receptors contain specific sequence motifs in their intracellular tails to recruit signaling molecules. The most common inhibitory motif is the immunoreceptor tyrosine-based inhibitory motif (ITIM). Engagement of ITIM-bearing receptors results in ITIM tyrosine phosphorylation and recruitment of downstream mediators containing Src homology 2 (SH2) domains, such as SHP-1, SHP-2, SHIP, and Csk [9]. Next, dephosphorylating signaling intermediates causes them to act on their respective targets to dampen inflammatory signals relayed by activating receptors. ITIM-containing receptors mostly, but not exclusively [3], attenuate immunoreceptor tyrosine-based activation motif (ITAM)–associated receptors, such as Fcγ receptors (FcγRs).

**Fig 2:**
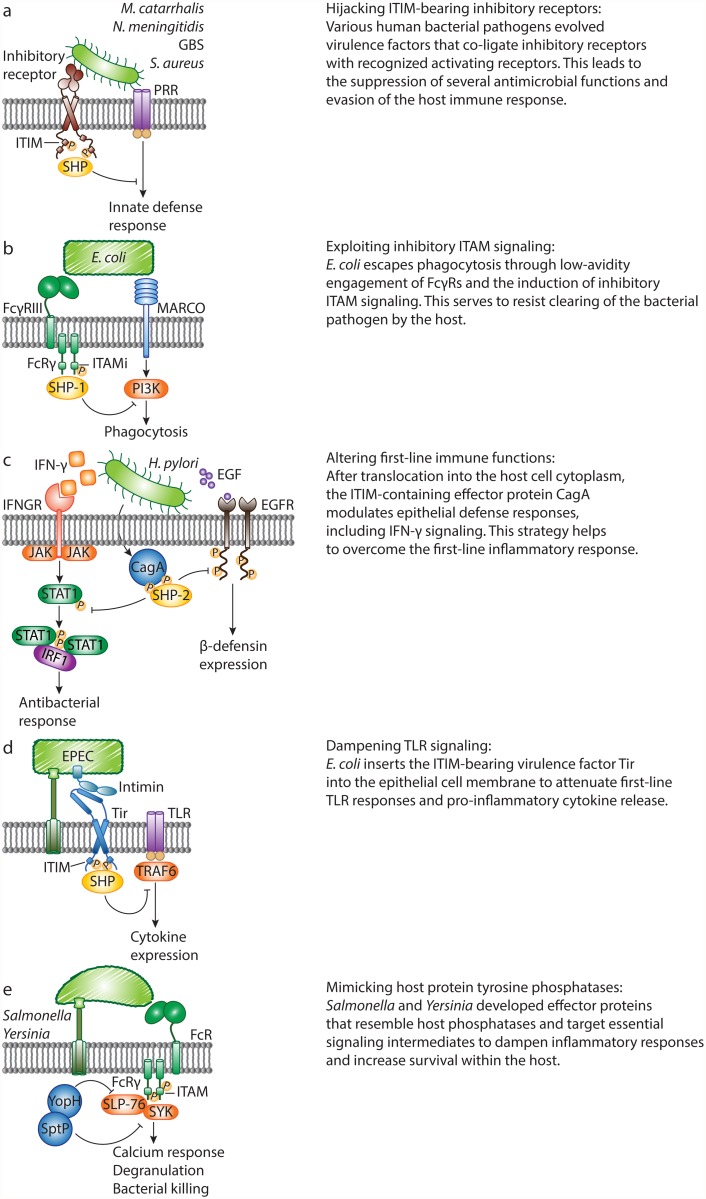
Bacterial pathogens evade host defense responses by manipulating inhibitory signaling. A. *M. catarrhalis*, *N. meningitidis*, Group B *Streptococcus* and *Staphylococcus aureus* evolved specific virulence factors to engage inhibitory receptors, which co-ligate with and attenuate pattern recognition receptor (PRR) signaling. B. *Escherichia coli* escapes macrophage receptor with collagenous structure (MARCO)–dependent killing through hijacking of inhibitory ITAM signaling. Non-opsonized *E. coli* binds to FcγRIII with low affinity and induces weak phosphorylation of the FcR common γ chain (FcRγ), leading to recruitment of SHP-1. In turn, SHP-1 dephosphorylates PI3K and abrogates MARCO-dependent phagocytosis. C. Upon infection, *Helicobacter pylori* translocates the ITIM-containing virulence protein, CagA, into host cells, and CagA-SHP-2 interactions lead to dephosphorylation of activated STAT1 and epidermal growth factor receptor (EGFR). This abrogates IFN-γ signaling and human β-defensin 3 (hBD3) synthesis, and enhances bacterial survival. D. During infection with the bacterium enteropathogenic *E. coli* (EPEC), the intimin receptor (Tir) translocates into the epithelial cell. The intracellular tail of EPEC Tir recruits host cell phosphatases SHP-1 and SHP-2. As a result, the activation of TRAF6 is inhibited, and EPEC-induced expression of pro-inflammatory cytokines is suppressed. E. *Salmonella* and *Yersinia* secrete protein tyrosine phosphatases SptP and YopH, respectively. SptP targets the protein tyrosine kinase SYK in mast cells and suppresses degranulation. During in vivo infection, YopH targets the signaling adaptor SLP-76 in neutrophils. This leads to reduced calcium responses and IL-10 production.

Undesirable outcomes may arise, however, when bacteria take advantage of host inhibitory signaling. Some bacterial pathogens use surface ligands to directly engage ITIM-bearing receptors, which they can co-ligate with activated PRRs (such as TLRs) or ITAM-paired receptors to suppress cellular activation and increase bacterial survival (Fig. 2a). For instance, following inhalation of *Moraxella catarrhalis* or *Neisseria meningitidis*, pulmonary epithelial cells release IL-8 and GM-CSF in a TLR-2-dependent manner, to recruit neutrophils. To evade immune clearance, the virulence proteins UspA1 of *M. catarrhalis* and Opa of *N. meningitidis* ligate the ITIM-containing receptor carcinoembryonic antigen-related cell adhesion molecule 1 (CEACAM1) with TLR-2 on epithelial cells, thus inhibiting co-engaged TLR-2 signaling and cytokine release [10]. Similarly, Nakayama et al. reported that *Staphylococcus aureus* targets the murine ITIM-bearing inhibitory receptor paired Ig-like receptor B (PIR-B) through the essential cell wall component lipoteichoic acid (LTA) to blunt TLR-induced inflammatory cytokine release by macrophages in response to the bacteria [5,11]. *S. aureus*, a major source of mortality in hospitals, can spread to the bloodstream and cause life-threatening sepsis. Following challenge with *S. aureus*, *Pirb*-knockout mice show enhanced inflammatory responses, and are better at clearing the bacteria and resistant to *S. aureus*-induced sepsis.

In addition, Group B *Streptococcus* (GBS) uses an evasion strategy to oppose ITAM-mediated inflammatory responses. The surface β-protein and sialic acid of GBS both suppress host defense by engaging inhibitory sialic acid–binding Ig-like lectins (Siglecs). GBS, an important cause of neonatal infections, targets ITIM-bearing Siglec-5 and Siglec-9 to recruit SHP-2 and escape killing by monocytes and/or neutrophils in vitro [12,13]. In line with these findings, mice lacking Siglec-E (the orthologue of human Siglec-9) clear GBS more quickly than wild-type mice [4]. Recently, the ITAM-coupled Siglec-14 was shown to counteract Siglec-5–dependent host immune suppression by GBS, thus forming a paired receptor system that balances inflammatory responses to bacterial pathogens [14]. Together, an increasing number of studies demonstrate that bacterial pathogens target ITIM-containing inhibitory receptors to suppress immune cell function and increase their survival within the host.

## ITAMs That Deliver Inhibitory Signals—And Their Manipulation by *E. coli*

Although ITAM motifs generally deliver activating signals, growing evidence now supports a role for ITAM-associated receptors in mediating inhibitory signals. ITAMs are found in the cytoplasmic domains of host receptors but also in certain transmembrane adaptors, such as the FcR common γ chain (FcRγ) in myeloid cells, that pair with specific receptors. ITAM-mediated cell activation requires high-avidity ligation of the ITAM-coupled receptors. In contrast, low-avidity ligation of these receptors generates inhibitory signals [15,16]. An ITAM that is functioning in an inhibitory mode is referred to as an “ITAMi”. Bacterial pathogens can hijack ITAMi signaling as a means to subvert defense responses. Specifically, *E. coli* binds FcγRIII directly in an antibody-independent manner, and this low-avidity interaction induces FcRγ phosphorylation, followed by SHP-1 recruitment (Fig. 2b). In turn, recruitment of SHP-1 is associated with a reduction in phosphorylation of PI3K, which is thereby unable to support MARCO-mediated phagocytosis of *E. coli* [17]. Consequently, mice deficient in FcγRIII or FcRγ have increased survival rates in models of sepsis, and this is attributed, in part, to their enhanced ability to clear *E. coli*. Thus, *E. coli* can overcome the inflammatory response through manipulation of the FcγRIII-FcRγ signaling complex, resulting in severe consequences during sepsis. To date, the identity of the FcγRIII-interacting ligand of *E. coli* remains unknown. Also, since these findings are not exclusive to pathogenic *E. coli*, it is conceivable that this crosstalk instead evolved to limit and control unwarranted inflammatory responses to commensal organisms, such as gram-negative bacteria in the gut.

## *H. pylori* and Enteropathogenic *E. coli* Hack into Host Inflammatory Signaling Through ITIM-Containing Effector Secretion

The above-mentioned examples include strategies where bacterial cell surface ligands directly interact with ITIM-bearing or ITAMi-paired host receptors to overcome host defenses. Successful bacterial pathogens may also use virulence factors that are delivered into host cells via specialized secretion systems and interfere with cellular signaling. These bacterial proteins are commonly referred to as “effectors”. Remarkably, recent studies have revealed that bacterial pathogens secrete effectors to relay inhibitory signals. Enteropathogenic *E. coli* (EPEC) and *H. pylori* release effectors containing ITIM-like motifs within target cells to suppress immune responses [8,18–20], while *Yersinia* and *Salmonella* attenuate inflammatory signaling through secretion of effectors that bear resemblance to host cellular protein tyrosine phosphatases (PTPases) [6,7].

The first identified bacterially encoded effector containing tyrosine-based motifs resembling ITIMs, is the major virulence factor cytotoxin-associated gene A (CagA) from *H. pylori*, a cause of gastric inflammation. During *H. pylori* infection, a type IV secretion system is formed that exports CagA into host cells. Translocated CagA undergoes tyrosine phosphorylation in the host cells and directly mediates SHP-2 activation by binding to SH2 domains in a phosphorylation-dependent manner [21]. Work by Wang et al. described that the effector CagA modulates epithelial cell inflammatory responses by preventing the induction of IFN-γ–dependent STAT1 phosphorylation and IRF1 transactivation in targeted epithelial cells (Fig. 2c) [8]. Similarly, *H. pylori* counteracts the expression of the antimicrobial defensin peptide human β-defensin 3 (hBD3), to which *H. pylori* is highly susceptible (Fig. 2c) [20]. Following activation by CagA, SHP-2 dephosphorylates the intracellular domains of EGFR, thereby abrogating hBD3 synthesis and increasing bacterial survival.

Bioinformatics approaches revealed that the bacterial effector translocated intimin receptor (Tir) of enterohaemorrhagic *E. coli* (EHEC) and EPEC encodes similar ITIM-like motifs [22]. EPEC uses a strategy where it injects bacterial Tir into the epithelial cell membrane (Fig. 2d). The extracellular part of Tir is engaged by the bacterial surface ligand intimin, while the intracellular part of Tir contains a region with similarity to host ITIMs. The bacterial Tir ITIMs recruit the host tyrosine phosphatases SHP-1 and SHP-2 [18,19], which enhance its binding to TRAF6. The resulting interaction inhibits the ubiquitination and activation of TRAF6 and thereby suppresses EPEC-induced expression of inflammatory cytokines.

## *Salmonella* and *Yersinia* Break Down Host Defense Responses through Bacterial PTPases

*Salmonella* and *Yersinia* contain effector proteins that resemble host PTPases and target central inflammatory signaling to shut down host immune cells (Fig. 2e) [6,7]. Like CagA and Tir, PTPase-like bacterial effectors were discovered by sequence homology studies [23]. The structurally related effectors SptP and YopH share homology with eukaryotic PTPases [23] and are essential for virulence of *Salmonella* and *Yersinia*, respectively. Bacterial PTPases have a myriad of known host targets and other well-established functions. In this review, we focus on the bacterial strategies that disrupt inflammatory responses through dephosphorylation of signaling intermediates by bacterial PTPases. We do not cover general tactics used by bacteria to interfere with inflammatory signaling through inactivation of signaling molecules by other effector proteins, such as the *Yersinia* leucine-rich repeat effector YopM [24,25]. Choi et al. demonstrated that *Salmonella typhimurium* secretes SptP to impede inflammatory responses [6]. SYK is an essential protein tyrosine kinase for IgE-mediated mast cell degranulation. WT *S. typhimurium* suppresses IgE-induced phosphorylation of SYK, whereas an isogenic Δ*sptP* mutant is not able to do so. In vivo, mast cells fail to degranulate and recruit neutrophils upon infection with *S. typhimurium*. In the absence of SptP, however, mast cells do degranulate and neutrophils are rapidly recruited to sites of infection, demonstrating a direct role for SptP in suppressing mast cell activation, neutrophil influx and bacterial clearance.

Neutrophils are also recruited to inflammatory lesions after infection with *Yersinia pseudotuberculosis* (*Yptb*), another gram-negative human pathogen. In a recent study, it was shown that the PTPase-like effector YopH affects phosphorylation of the crucial signaling adaptor SLP-76 and activation of its downstream effectors in recruited neutrophils, dampening calcium responses and IL-10 production [7]. Depletion of neutrophils allows the outgrowth of a mutant lacking YopH, indicating that YopH is critical for attenuating neutrophil bactericidal functions to enhance survival of *Yptb*.

## Concluding Remarks

Since the first descriptions of an ITIM in FcγRIIB and CD22 over 15 years ago [26,27], many inhibitory receptors are still being discovered by the presence of intracellular inhibitory motifs. To date, genomic and proteomic informatics revealed more than 300 ITIM-bearing proteins [28,29], and many of them still await demonstration of function. Studying the mechanisms of bacterial manipulation of inhibitory signaling may provide useful insights on the functional roles of novel ITIM-bearing receptors. Clearly, bacteria have evolved sophisticated strategies to successfully instigate host inhibitory signaling, allowing evasion of immune defense mechanisms. Insight regarding these strategies is crucial to design approaches to control infection. In an era of growing resistance to antibiotics, blocking of subverted host receptors or counteracting the virulence factors involved affords promising approaches to overcome immune evasion by pathogens.
