# Correction: Bacterial Immune Evasion through Manipulation of Host Inhibitory Immune Signaling

**DOI:** 10.1371/journal.ppat.1004813

**Published:** 2015-04-08

**Authors:** The PLOS Pathogens Staff

The second author’s name is incorrect in the citation. The correct name is: van Sorge NM.

The correct citation is:

Van Avondt K, van Sorge NM, Meyaard L (2015) Bacterial Immune Evasion through Manipulation of Host Inhibitory Immune Signaling. PLoS Pathog 11(3): e1004644. doi:10.1371/journal.ppat.1004644

## References

[1] Van Avondt K, Sorge NMv, Meyaard L (2015) Bacterial Immune Evasion through Manipulation of Host Inhibitory Immune Signaling. PLoS Pathog 11(3): e1004644 doi: 10.1371/journal.ppat.1004644 25742647 10.1371/journal.ppat.1004644 PMC4351076

